# Increased nicotine response in iPSC-derived human neurons carrying the CHRNA5 N398 allele

**DOI:** 10.1038/srep34341

**Published:** 2016-10-04

**Authors:** Eileen N. Oni, Apoorva Halikere, Guohui Li, Alana J. Toro-Ramos, Mavis R. Swerdel, Jessica L. Verpeut, Jennifer C. Moore, Nicholas T. Bello, Laura J. Bierut, Alison Goate, Jay A. Tischfield, Zhiping P. Pang, Ronald P. Hart

**Affiliations:** 1 Cell Biology and Neuroscience, Rutgers University, Piscataway, NJ, USA; 2 Child Health Institute of New Jersey & Dept. of Neuroscience and Cell Biology, Rutgers Robert Wood Johnson Medical School, New Brunswick, NJ, USA; 3 Department of Animal Sciences, Rutgers University, New Brunswick, NJ, USA; 4 Human Genetics Institute of New Jersey, Rutgers University and RWJMS, Piscataway, NJ, USA; 5 Department of Human Genetics, Rutgers University, Piscataway, NJ, USA; 6 Department of Psychiatry, Washington University School of Medicine in St. Louis, St. Louis, MO, USA; 7 Neuroscience Department, Icahn School of Medicine at Mount Sinai, New York, NY, USA.

## Abstract

Genetic variation in nicotinic receptor alpha 5 (*CHRNA5*) has been associated with increased risk of addiction-associated phenotypes in humans yet little is known the underlying neural basis. Induced pluripotent stem cells (iPSCs) were derived from donors homozygous for either the major (D398) or the minor (N398) allele of the nonsynonymous single nucleotide polymorphism (SNP), rs16969968, in *CHRNA5*. To understand the impact of these nicotinic receptor variants in humans, we differentiated these iPSCs to dopamine (DA) or glutamatergic neurons and then tested their functional properties and response to nicotine. Results show that N398 variant human DA neurons differentially express genes associated with ligand receptor interaction and synaptic function. While both variants exhibited physiological properties consistent with mature neuronal function, the N398 neuronal population responded more actively with an increased excitatory postsynaptic current response upon the application of nicotine in both DA and glutamatergic neurons. Glutamatergic N398 neurons responded to lower nicotine doses (0.1 μM) with greater frequency and amplitude but they also exhibited rapid desensitization, consistent with previous analyses of N398-associated nicotinic receptor function. This study offers a proof-of-principle for utilizing human neurons to study gene variants contribution to addiction.

Drug abuse and addiction are a major burden to society with the total cost of substance abuse in the U.S. exceeding $600 billion annually (NIDA). Smoking is the largest preventable cause of death in the U.S., leading to more than 465,000 deaths in 2010^1^. Since addiction and comorbid behaviors are a consequence of brain function, the understanding of the neural basis underlying addiction becomes crucial to determine the etiology and the pathophysiology of addiction. However, little is known about the specific neuronal, synaptic and cell biological mechanisms underlying addiction behaviors, impacting the variable treatment outcomes for substance abuse disorders.

Nicotine, considered to be the primary contributor to tobacco addiction, stimulates the nicotinic acetylcholine receptor (nAChR) of different neuronal subtypes in the brain including the midbrain dopaminergic (DA)^2^,^3^ as well as forebrain glutamatergic neurons^4^,^5^. Genome wide association studies (GWAS) have identified several genetic variants associated with increased risk of addiction, including single nucleotide polymorphism (SNP) variants within the *CHRNA5-CHRNA3-CHRNB4* gene cluster of the 15q25 region, encoding the α5, α3, and β4 subunits of nAChR^6^,^7^. Of these variants, the SNP rs16969668 encodes an amino acid substitution at position 398 from aspartate (D) to asparagine (N), referred to as the D398 (major) or N398 (minor) alleles, of *CHRNA5*. Given that variation in α5 expression alters nAChR expression and function^8^, nonsynonymous variants within α5 are likely to affect neurophysiological functions, which potentially contribute to addictive behaviors^6^. However, how such nAChR gene variants in humans affect receptor activity along with neuronal function and lead to addiction behaviors is not known.

Studies using animal models provide some clue for how nAChR may contribute to addiction behavior. The α5 knockout mouse models exhibit increased nicotine consumption compared to controls, suggesting a role for the α5 subunit in modulating reward behavior^9^,^10^. The N398 α5 subunit, when selectively expressed in VTA (ventral tegmental area) DA neurons of CHRNA5^−/−^ mice, produced a partial loss of function and increased nicotine self-administration^11^. Mechanistic studies using similar systems concluded that the functional change in N398 is likely due to intracellular modulation of the receptor^12^. Meanwhile, heterologous expression of the human N398 α5 variant in Xenopus oocytes revealed it might affect the expression of the other nAChR subunits^13^, reduce Ca^2+^ permeability and increase short-term desensitization to nicotine^14^.

While carefully controlled animal models have been quite successful in understanding the global mediating pathways of motivational and reward behaviors, attempts to study more complex human diseases in animal models may correlate poorly, given the evolutionary distance between humans and rodents, as has been demonstrated in inflammation models^15^, spinal cord injury^16^, and neurodevelopmental disorders^17^. The mouse-human evolutionary divergence is further illustrated through the report of a human-specific nicotinic acetylcholine receptor (*CHRFAM7A*) that is thought to play a role in inflammatory response^18^. Given the complexity of human neuronal function compared to that of rodents, it is critical to understand the role of nAChR gene variants in modulating neuronal function in a human-specific context. To overcome the lack of living human neurons to study the role of human nAChR genetic variants, recent advances in stem cell biology, especially the development of subject-specific induced pluripotent stem cells (iPSCs) now allows the use of human neurons derived from phenotypically variant subjects to model human neuropsychiatric disorders at a cell biological and mechanistic level, which was not previously possible^19^,^20^,^21^,^22^,^23^.

Here we have drawn upon the collection of cryopreserved lymphocytes stored in the Collaborative Genetic Study of Nicotine Dependence (COGEND) repository to construct multiple induced pluripotent stem cell (iPSC) subject lines with and without the α5 N398 variant using non-integrating Sendai viral vectors^24^,^25^. We successfully derived two subtypes of neurons from these iPSCs: (1) midbrain DA neurons using a dual-SMAD inhibition protocol^26^; as well as (2) pure excitatory forebrain glutamatergic neurons generated using induced neuronal (iN) technology^27^. We found that DA neurons exhibit mature neuronal characteristics such as spontaneous repetitive action potentials (APs) and synaptic activities; expressing pan-neuronal markers, midbrain DA neuronal markers, and nAChR mRNAs. Gene ontology (GO) and pathway analysis of RNAseq data as well as functional characterization using electrophysiology revealed functional and nicotine response differences between N398 and D398 neuron cultures. Interestingly, our results indicate that the DA neurons also co-release glutamate. The N398 DA neurons exhibit a more profound response to nicotine stimulation and this response is likely caused by presynaptic input from glutamatergic neurons in the culture. Moreover, nicotine exhibited a transiently increased but desensitizing effect on synaptic transmission in excitatory iN cells. These results provide important insights on how *CHRNA*5 gene variants may affect neuronal activity and offer a proof-of-principle for utilizing human neurons derived from somatic cells to study the contribution of gene variants to addiction behavior.

## Results

### Generation of iPSCs from blood cells of multiple subjects carrying CHRNA5 D398/N398 gene variants

**Figure 1:**
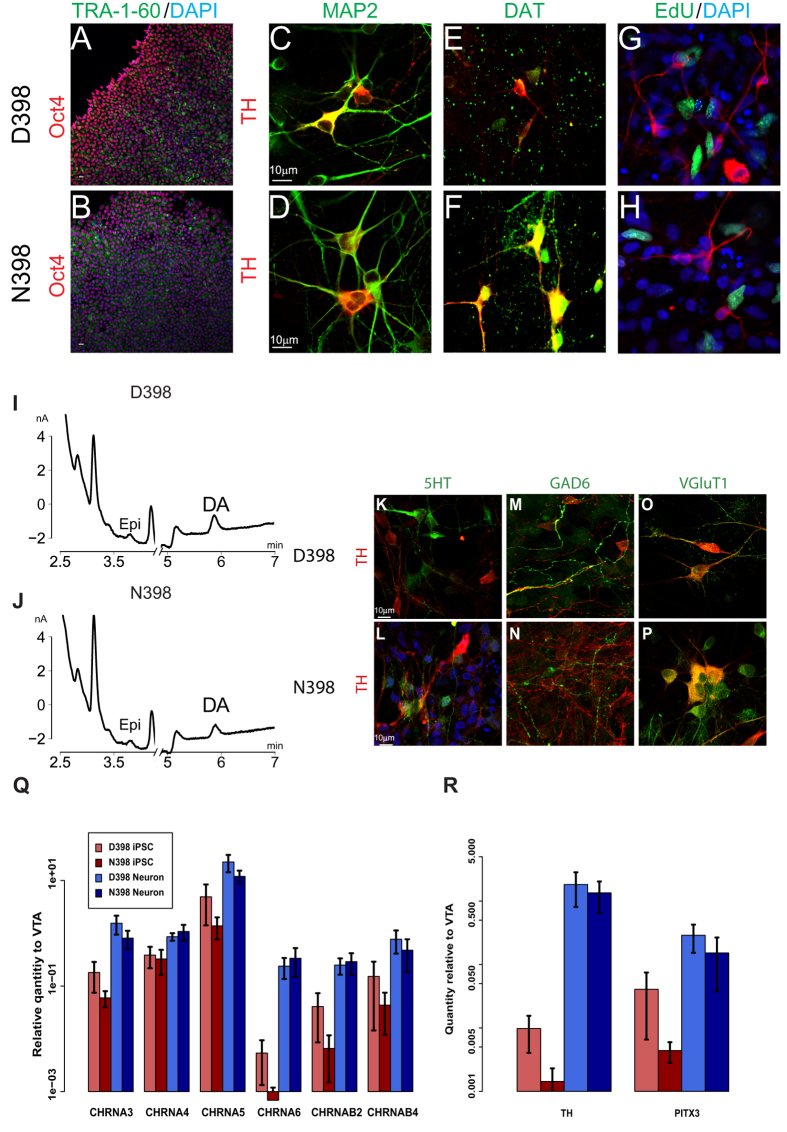
Functionally mature midbrain-like DA neurons present in both D398 and N398 DA cultures. Representative iPSC cultures from (**A**) D398 and (**B**) N398 groups stained positively for pluripotency markers TRA-1-60 (green) and Oct4 (red). The majority of cells in iPSC-derived DA cultures expressed both MAP2+ (green) cells and TH (red) in both D398 (**C**) and N398 (**D**) groups. (**E,F**) Punctate staining indicates the presence of DAT (green), likely localized to synapses and surrounding TH+ cells. (**G,H**) Few TH+ were labeled with a pulse of EdU (green), indicating that most cells were postmitotic. (**I,J**) HPLC traces showing release of DA into culture medium with 1 mM nicotine. (**K–P**) Identification of alternative neuronal subtypes in mDA cultures. Cultures were stained with TH (red) and either (**K,L**) 5HT, (**M,N**) GAD6, or (**O,P**) VGluT1 (green). (**Q**) qPCR analysis of nAChR subunit-encoding mRNAs a3,a4,a5,a6, b2, b4, of D398 (red) or N398 (blue), for either iPSC (lighter) or DA (darker) cultures. mRNA levels are normalized to GAPDH and human fetal VTA RNA. Cultures were also assayed by qPCR for mRNAs encoding (**R**) TH and the midbrain marker PITX3. For all qPCR assays, DA neurons were different from iPSC cultures (ANOVA, p<0.05) but there was no difference between N398 and D398.

Nicotine dependent subjects were selected from the COGEND collection^6^,^28^ using the results of the Fagerström test of nicotine dependence^29^ and the homozygous presence of the minor allele (AA) of rs16969968 as well as the presence of homozygous major alleles for other SNPs known to affect addiction behaviors and/or nAChR function: rs880395 (GG), rs8192475 (GG), rs12914008 (GG), and rs56218866 (TT) (Supplemental Table 1). Non-nicotine dependent control subjects who smoked came from the same collection, and were homozygous for the major allele of rs16969968 (GG) but matched at the other SNPs. One male and two females were selected for each group. Primary lymphocytes were processed for reprogramming as described previously^24^. Pluripotency was confirmed by immunocytochemistry (ICC) for Oct4 and TRA-1-60 (Fig. 1A,B), by embryoid body formation followed by detection of three germ layers by immunocytochemistry (not shown), and by gene expression studies, including the PluriTest algorithm^30^ (Supplemental Table 2). All tests were positive for pluripotency.

### CHRNA5 N398 DA neurons exhibit greater induced activity

To study the functional consequences of the N398 genetic variant of *CHRNA5*, we used each of the six subject-derived iPSC lines to generate human DA neuron cultures resembling midbrain VTA DA neurons, which are critical mediators of the mesolimbic DAergic system, a pathway whose performance is altered in addiction^31^,^32^. iPSC were differentiated using the dual SMAD inhibition protocol^26^. Expression of DA-specific and mature neuronal markers was confirmed by ICC staining of the rate-limiting enzyme tyrosine hydroxylase (TH), the early neuronal marker TuJ1 (βIII tubulin, not shown), and the more mature neuronal marker MAP2 (Fig. 1C,D). More than 75% of MAP2-labeled cells coexpressed TH (D398 75.0% ± 0.092, n = 3; N398 78.4% ± 0.027, n = 3). Cells positive for TH were also associated with punctate staining for the vesicular DA transporter (DAT; Fig. 1E,F). To confirm that TH^+^ cells were postmitotic, cultures were incubated with EdU for 24 hours to label cells in S phase. Only a small fraction of TH^+^ cells stained positive for EdU (Fig. 1G,H; 3.0 ± 0.007% for D398; 2.02 ± 0.008% for N398, n = 3), indicating that nearly all cells were postmitotic.

To determine whether the cultures were capable of releasing DA in response to stimulation, we replaced culture medium with buffer containing 1 mM nicotine for 30 minutes, and analyzed the DA content in the medium using HPLC. A distinct peak consistent with DA retention time was observed (Fig. 1I,J). All results are consistent with the presence of midbrain-like DA neurons, but the cultures also contained a small minority of serotonergic (5-HT positive) and inhibitory (GAD6 positive) neurons (Fig. 1K–N). Results also identified cells that were both TH^+^ and VGluT^+^ (Fig. 1O,P), consistent with the notion that some DA neurons also co-release glutamate^33^,^34^. Moreover, DA cultures express mRNAs encoding nAChR subunits α3, α4, α5, α6, β2, and β4 at levels similar to those found in developing midbrain VTA, using a sample of human fetal VTA mRNA as a reference (Fig. 1Q). To further confirm the lineage of our culture, we observed mRNA expression for TH and the classic midbrain marker, PITX3 (Fig. 1R). All nAChR subunit and marker mRNAs increased significantly upon differentiation from iPSC to midbrain-like DA neuron cultures (ANOVA, p < 0.05) even though there was some variability between iPSC lines derived from different subjects, as depicted by the large error bars shown in Fig. 1Q,R. However there was no significant difference in expression between *CHRNA5* variant groups. Therefore, the rs16969968 SNP in *CHRNA5* does not affect the generation of the DA cellular phenotype through the dual SMAD inhibition protocol.

**Figure 2:**
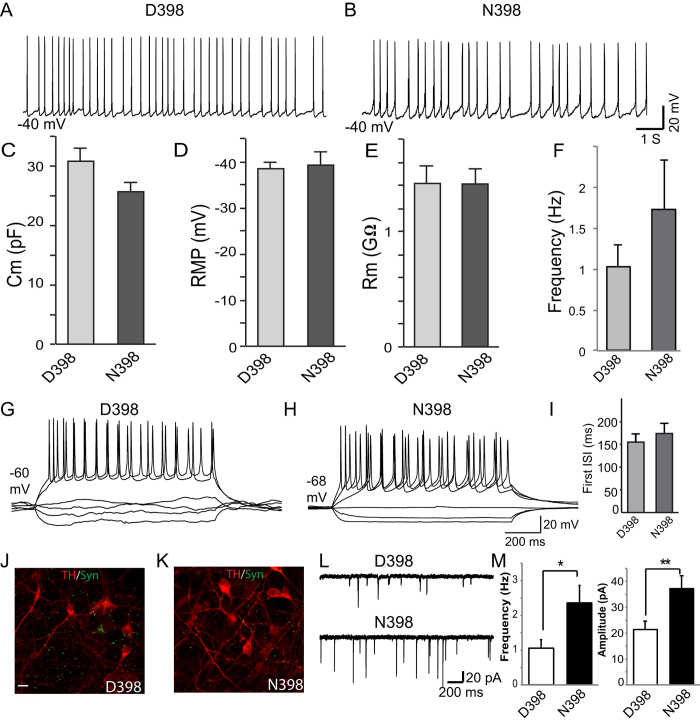
Spontaneous postsynaptic activity is increased in N398 DA cultures. (**A**,**B**) Spontaneous action potentials of D398 and N398 DA neurons. (**C**) Membrane capacitance, (**D**) Resting membrane potentials (RMP), (**E**) Input resistance of cell membrane (Rm), and (**F**) Spontaneous firing frequency of D398 and N398 DA neurons. (**G**,**H**) Repetitive action potentials from depolarizing current injections in D398 and N398 DA neurons. (**I**) Interstimulus intervals of induced action potentials of D398 and N398 DA neurons. (**J**,**K**) ICC of TH (Red) and Synapsin (Green) for D398 and N398 DA neurons. (**L**) Spontaneous postsynaptic currents of D398 and N398 DA neurons. (**M**) Frequency and amplitudes of postsynaptic current responses from D398 and N398 DA neurons.

We next assessed the functional parameters of derived human DA neurons from subjects with D398 or N398 variants. Using whole cell patch clamp recordings, we found that both the D398 and N398 DA cultures produced robust and repetitive spontaneous action potentials (APs) (Fig. 2A,B) and had similar membrane properties, including membrane capacitance, resting membrane potentials, and input resistance (Fig. 2C–E). While the spontaneous APs in DA neurons of both genotypes have statistically similar frequencies (p = 0.258), the N398 neurons tend to fire more APs (Fig. 2F). Next, we tested the intrinsic excitability of these DA neurons by inducing APs using different amplitudes of current injections; however, neurons of both genotypes exhibited similar neuronal excitability as indicated by a similar frequency of induced APs (Fig. 2G,H). These data suggest that the N398 variant in *CHRNA5* does not impair neuronal excitability, nor does it produce any major changes in the passive membrane properties of D398 and N398 human neurons (Fig. 2I). However, while DA neurons with different *CHRNA5* gene variants have similar intrinsic excitabilities, the N398 variants also have a slightly higher frequency of spontaneous APs (Fig. 2F), which could be due to differences in synaptic network activity. Since the DA neuronal cultures also form synapses as revealed by synapsin puncta (Fig. 2J,K), we turned to measurements of postsynaptic activity.

In order to test whether the N398 variant alters synapse function, we measured spontaneous postsynaptic currents (sPSCs, Fig. 2L). Both N398 and D398 neurons exhibited robust sPSCs, which is indicative of mature synapse formation and synaptic transmission among these neurons. Interestingly, consistent with the slightly greater spontaneous AP firing frequency in N398 DA neurons, the N398 DA neurons also exhibited greater spontaneous postsynaptic synaptic activity when compared to the D398 neurons (Fig. 2L,M), indicated by the increased frequency and the amplitudes of the sPSCs in N398 neurons when compared to the D398 DA neurons. These results were supported by postsynaptic electrophysiological analysis in response to 3 μM nicotine stimulation (Supplemental Fig. 1). Results illustrate that D398 DA neurons respond variably to nicotine exposure, but more N398 neurons seemed to be potentiated by nicotine. Therefore, neurons in the N398 cultures have a higher excitability due to more synaptic activity.

### Gene expression patterns predict functional differences between D398 and N398 DA neuronal cultures

**Figure 3:**
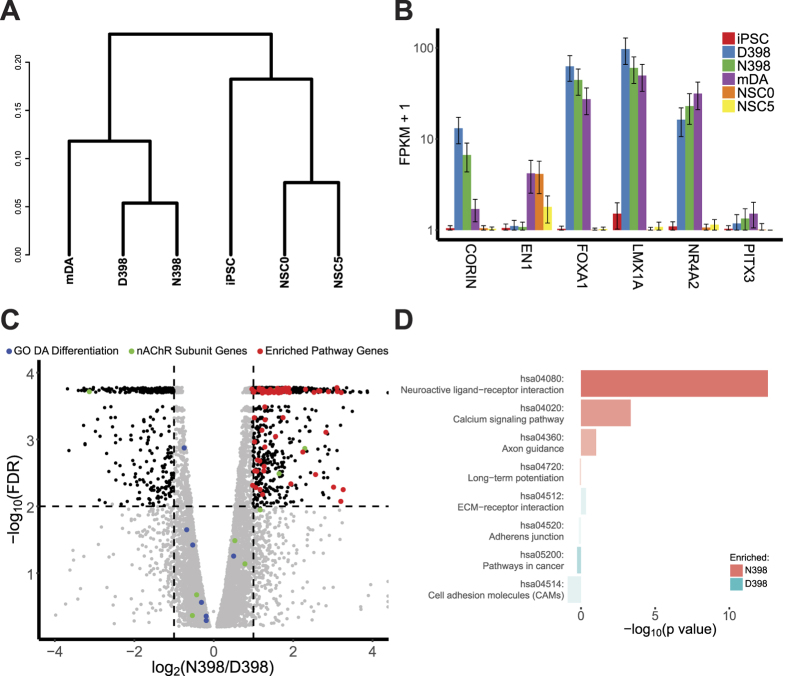
Gene expression differences in iPSC derived DA neurons exhibit patterns of differential response. (**A**) Hierarchical clustering indicates that D398 and N398 DA cultures were more similar to mDA cultures than to iPSC or iPSC-derived NSC (NSC0), or NSC differentiating into glutamatergic neurons (5 days of differentiation, NSC5). The dendrogram shows relative distances (based on the Jenson-Shannon divergence metric) for the top 500 genes varying by group. (**B**) Expression patterns for genes characteristic of midbrain DA neurons. The mean FPKM is plotted ± the 95% confidence interval, n = 3/group. (**C**) RNAseq volcano plot. The horizontal axis shows the log_2_ fold-change, comparing N398 to D398. The vertical axis shows the false discovery rate (FDR) as the −log_10_ of the value. Points are plotted as individual genes not significantly different (grey dots) or significantly different (black dots) at 1% FDR (horizontal dashed line) and at least 2-fold different (vertical dashed lines). Colored dots indicate the nAChR receptor neuronal markers (green), GO dopamine differentiation genes (blue) and differentially-regulated genes belonging to the significantly-enriched pathways relatively increased in N398 (red). (**D**) Pathway (KEGG) analysis identifies three functional groups as enriched in the list of genes increased in N398. Bar colors indicate enrichment in N398 (red) or D398 (blue). The bar length indicates the FDR, and the color intensity (alpha) indicates the number of genes enriched. See Supplemental Table 4 for additional functional analyses.

In order to probe the gene expression network changes that may contribute to the functional differences we observed between D398 and N398 human neurons (Fig. 2), we conducted gene expression profiling by RNAseq, using replicate cultures from single donors from each variant group (Fig. 3). We included in our analysis RNAseq data from control DA cultures published by the Studer lab (mDA)^35^, iPSC cultures, and hESC-derived neural stem cells (NSC) at two stages of differentiation (producing glutamatergic neurons, day 0 [NSC0] and day 5 [NSC5])^36^. Clustering analysis shows that D398 and N398 cultures are more similar to DA neuron cultures than to cortical glutamatergic NSC (Fig. 3A). We find similar expression levels of several mRNAs known to be associated with DA neurons in midbrain-like DA, D398, and N398 cultures (Fig. 3B). *CORIN*, *FOXA1*, *LMX1A*, *NR4A2*, and *PITX3* mRNAs are all prominent and similar to levels found in previously-published mDA cultures. *EN1* levels are lower, but well above the limit of detection, and were also observed in cultures of excitatory neuron precursors (NSC0 and NSC5). Expression plots for several other groups of midbrain-specific and contrasting cortical-specific genes are depicted in Supplemental Fig. 2A–G. All results support the relationship shown in Fig. 3A, that the N398 and D398 cultures are most similar to the previously-published mDA cultures. We conclude that gene expression patterns are consistent with the presence of midbrain-like DA neurons in our cultures.

The vast majority of mRNAs detected in these cultures was expressed at similar levels in either D398 or N398 DA neurons. The set of nAChR subunit mRNAs (Fig. 3C, green dots), as previously detected by qPCR (Fig. 1Q), was primarily within the 2-fold difference range (depicted by the vertical dashed lines), except for *CHRNA5*, which was relatively increased in D398 (8.7-fold higher, 0.02% FDR). qPCR assay (Fig. 1Q) indicated a similar difference although it was not statistically significant. Subjects were chosen for a homozygous major allele in rs880395 to exclude the potential effect on CHRNA5 mRNA levels observed previously^37^. Other groups of genes indicative of neuronal identity or maturity, including the gene ontology group members specific for dopaminergic differentiation (Fig. 3C, blue dots; also see Supplemental Fig. 3) were unaffected by the variation.

Focusing on differences between D398 and N398 cultures, 1,194 genes were significantly different (≤1% FDR and at least 2-fold different, dashed lines; black dots in Fig. 3C). Taking the 349 genes that were relatively lower in N398, only two KEGG pathways were found to be enriched at relatively low confidence levels (blue bars in Fig. 3D), as determined by DAVID software^38^,^39^. However, the 845 genes relatively increased in N398 were significantly enriched in Neuroactive ligand-receptor interaction, Ca^2+^ signaling pathway, and Axon guidance KEGG pathways (red bars in Fig. 3D). A selected sample of genes in this group is plotted in Supplemental Fig. 4H. Enrichment of GO functional groups similarly highlights neuron activity-specific biological processes (Supplemental Table 4). This predicts that N398 DA neuron cultures are likely to exhibit functional differences in response to ligand, potentially through changes calcium influx. This also suggests that the N398 variant may contribute to the excitatory response of neurons during nicotine exposure.

### Glutamatergic N398 neurons exhibit increased activity following nicotine exposure

**Figure 4:**
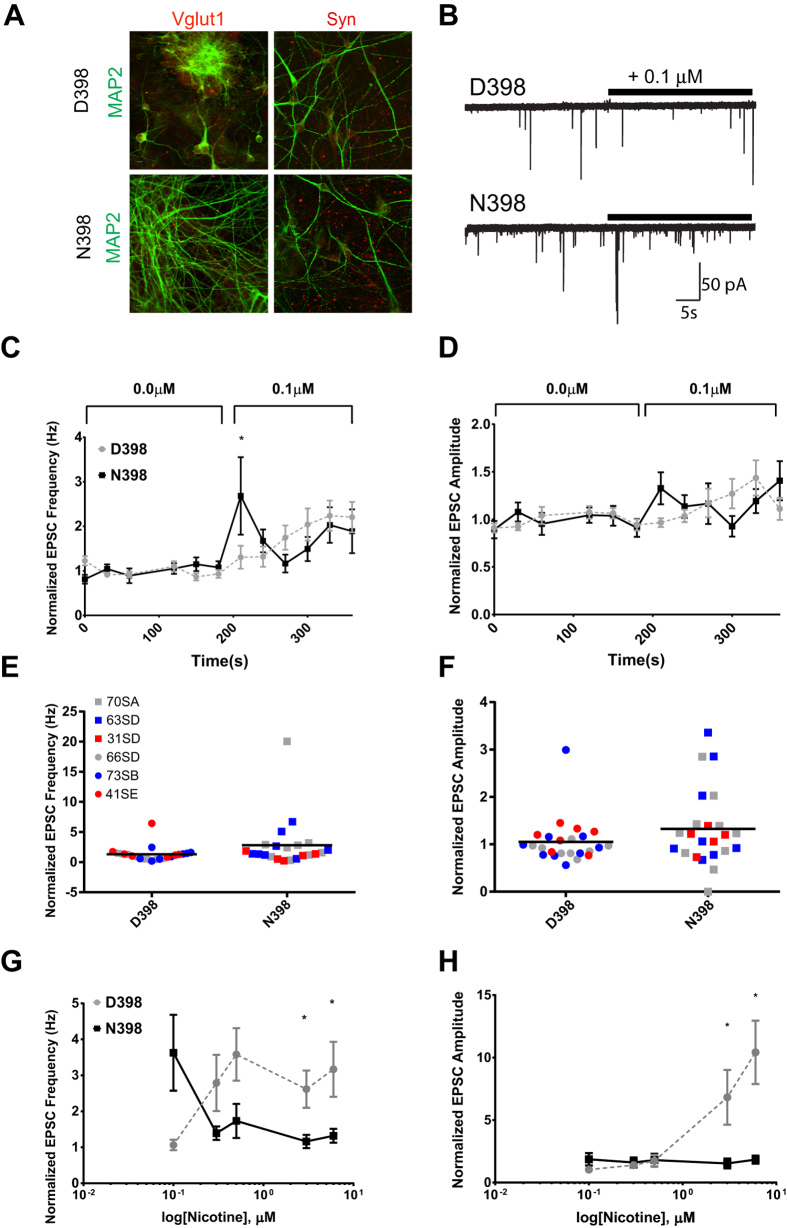
Excitatory N398 neurons exhibit increased response to nicotine followed by desensitization. (**A**) Glutamatergic iN cultures are positive for MAP2 (green), vesicular glutamate transporter 1 (VGluT1, left, red), and synapsin (Syn, right, red) as detected by ICC. (**B**) Example traces from a patched neuron to show changes in response upon introduction of 0.1 μM nicotine (indicated by bar). Patched neurons exhibited increased frequency (**C**) and amplitude (**D**) during initial exposure to 0.1 μM nicotine. By 60 s after nicotine addition, frequency trended lower in N398 cells, although not significantly. Plotting the initial response to 0.1 μM nicotine for individual cells, it is clear that the changes in frequency (**E**) or amplitude (**F**) in N398 was not due to a single cell or subject. D398 samples are plotted as circles and N398 as squares. Color denotes cells from individual subjects (see key and Supplemental Table 1). However, subsequent additions of increasing doses of nicotine had reduced initial frequency (**G**) and amplitude (**H**) in N398 compared with D398 cells. N398 was different in frequency response from D398 as assessed by a Tukey post-hoc test of a general linear mixed-effects model with repeated measures (p = 0.00046, n = 115 cells per genotype; 5 cells per culture; 5–7 cultures per individual cell line).

Results from the dual-SMAD-derived DA cultures suggest that human DA neurons may co-release glutamate (Fig. 1O,P) and the release of glutamate was likely differentially affected by the N398 or D398 nAChR variants (Fig. 2L,M). Gene expression patterns predict a difference in neurotransmitter response pathways, particularly for glutamate signaling. We considered whether the difference in response between N398 and D398 variant nAChRs could be observed in cultures of purely excitatory neurons, which have been puromycin-selected to increase the uniformity of the cultures. To test this, we differentiated N398 and D398 iPSC into forebrain excitatory neurons using iN protocols^27^ (Fig. 4A) and studied the impact of nicotine on synaptic transmission. Forebrain excitatory glutamatergic neurons are modulated by nAChRs^2^; thus we hypothesize that the N398 variant would have a greater functional impact on excitatory neurons. We examined the effects of nicotine on spontaneous excitatory PSCs (sEPSCs) in both D398 and N398 human excitatory neurons. The frequencies of sEPSCs in both N398 and D398 neurons are stable before the application of nicotine (Fig. 4B–D). However, within 30 s of 0.1 μM nicotine exposure, frequencies of sEPSCs were significantly increased in the N398 subjects compared to the D398 controls (Fig. 4C). Interestingly, following the dramatic increase of the sEPSCs in N398 neurons, the facilitatory effects appeared to decrease over time in the continued presence of nicotine, likely due to the desensitization of the receptor, which mimics the desensitization reported with ectopic expression studies in Xenopus oocytes^14^. Similarly, the amplitude of response trended higher during the initial 30 s but then fell below D398 shortly thereafter (Fig. 4D). The difference in response at 0.1 μM nicotine was not limited to a single outlier cell or to cells prepared from a single subject (Fig. 4E,F). The distribution of responses was variable but showed clearly that multiple cells from multiple subjects exhibited increased EPSC frequencies and amplitudes.

Since the greatest effect of nicotine on EPSCs occurred immediately after the application of nicotine, we analyzed responses from each cell during the initial 10 s exposures to sequential additions of 0.1 to 6 μM nicotine (Fig. 4G,H). The apparent desensitization in N398 was maintained during addition of higher doses of nicotine to the same patched cells. The amplitudes increased notably in D398 at higher doses (3 and 6 μM) while the N398 neurons exhibited minimal changes in frequency or amplitude of response. The combined effect of nicotine dose and *CHRNA5* genotype on EPSC frequency was significant overall at p = 0.00046 using a Tukey post-hoc test of an ANOVA linear mixed-effects model with repeated measures (n = 115/group, with a minimum of 25 cells in at least 3 different cultures from each of 3 different subjects per group). These results show that N398 neurons have higher initial responsiveness to nicotine but with enhanced desensitization. These data are largely in line with the notion that was reported previously using a heterologous expression system^14^. Therefore, the enhanced excitatory response and subsequent desensitization at concentrations of nicotine on par with that of heavy smokers carrying the N398 minor allele gene variant might contribute to their addictive behavior.

## Discussion

Our results indicate that the N398 variant of *CHRNA5* is associated with greater excitability and desensitization in response to nicotine, at least in excitatory cells but also affects the excitability of DA cells. The presence of rs16969968 minor allele in *CHRNA5* increases risk of nicotine addiction^6^,^7^. Experiments using heterologous expression of *CHRNA5* suggest that the N398 variant alters Ca^2+^ permeability and mediates nicotine consumption^9^,^10^,^11^,^14^,^40^. While these findings reveal potential effects on reward circuitry, as we began this study, we hypothesized that subtle effects of the α5 subunit genetic variants of nAChR would be detectable at the cellular level, especially at the synaptic level.

To address this hypothesis, we obtained repository blood samples collected as part of a large genetics study (COGEND) previously used for genome-wide association studies^6^,^28^. iPSC were prepared from cryopreserved, de-identified repository lymphocyte specimens. This strategy is particularly appealing because it allows access to large numbers of genetically diverse samples large from studies with cryopreserved specimens each having a clear clinical diagnostic history. We have developed reliable and reproducible methods to convert cryopreserved primary T-cells isolated from the RUCDR Infinite Biologics™ cell repository to iPSC^24^, and we successfully used this approach to study effects of alcohol on NSCs^41^ and mutations found in Ataxia telangiectasia^42^. We selected donors carrying homozygous rs16966968 major or minor allele, encoding the D398 or N398 variant, respectively, of the nAChR α5 subunit. Samples also contained homozygous major alleles for several related SNPs (Supplemental Table 1), particularly rs880395, since it has been reported to have secondary effects on CHRNA5 mRNA levels^37^. The goal was to represent isolated genotypes in order to test the difference between the major and minor allele of rs16969968. It has been suggested that cell lines from multiple patients and controls as well as multiple subclones from each patient should be analyzed to provide a convincing cellular or functional phenotype. However, the resulting high workload is a major obstacle to the identification of disease-associated phenotypes^43^,^44^,^45^. The most convincing solution to this problem is the creation of isogenic pairs of cells differing only in a single locus, usually by genetic engineering techniques^46^,^47^. Indeed, our previous RNAseq analyses demonstrate broad variability among subject iPSCs, even among members of the same family, and a vastly reduced variability when comparing isogenic samples^42^. However, editing to create an isogenic rs16969968 iPSC is time consuming and technically challenging. Nevertheless, our results derived from different subjects and different subtypes of human neuronal population are consistent and possibly more relevant overall to human populations. Therefore, we believe that by testing a reasonable number of subject-specific iPSC lines we capture sufficient variability while searching for reproducible phenotypes based on the non-synonymous SNP.

To begin to test *CHRNA5* variant function, midbrain-like DA neuronal cultures were chosen to mimic the DA neurons of the VTA, thought to be an important member of the reward/addiction circuit^31^,^32^. The presence of the N398 variant does not interfere with the generation of mature DA neurons, as determined by the expression of neuronal markers (Fig. 1), or of the basic membrane properties, and the capacity for generating action potentials is not different between the two *CHRNA5* variants (Fig. 2). Expression of nAChR mRNAs was similarly expressed in both variants. While mRNA levels cannot predict cellular protein levels or assembly into functional receptors, and a complete set of nAChR subunit-selective antibodies is not available, cultures expressed robust levels of mRNAs encoding *CHRNA3, 4, 5,* and *6*, *CHRNB2* and *4* as would be expected for central nAChRs (Fig. 1Q). We used fetal human VTA RNA as a reference, so we can conclude that the relative quantities of these mRNAs in cultures were within 2-3-fold of that found in developing midbrain DA neurons. However, it appears that N398 neurons are receiving more synaptic inputs, as revealed by an increased PSC amplitude and frequency in N398 compared with D398 (Fig. 2M), consistent with a possible increase in excitability of N398 neurons, or reduced input from inhibitory neurons, as suggested by the relative higher frequency of spontaneous action potentials. This correlates with results from others suggesting that while the α5 subunit does not contribute to nicotine binding with nAChRs, the loss or alteration of α5 contributes to a shift in nicotine response, specifically in VTA DA neurons^11^. We ascribed the increased network activity to interactions among the mixtures of neuronal subtypes in the culture. The dual-SMAD inhibition method was used to generate cultures of relatively high purity DA neurons (≥75% of MAP2^+^ cells co-expressed TH) with a smaller number of other neuron subtypes (Fig. 1K–P).

To investigate intracellular regulatory processes, we interrogated global gene expression by RNAseq. Results identified mRNAs differentially expressed between D398 and N398 (Fig. 3C and Supplemental Table 3). The most informative and significant functional enrichment was for KEGG pathways specific for neuroactive ligand receptor interaction, calcium signaling, and axon guidance (red bars in Fig. 3D). Whether the altered expression of these genes is an effect of the N398 variant or whether it is only a correlation due to differences in neuronal function, the RNAseq results demonstrate identifiable differences between cultures based on *CHRNA5* genotype and they predict that N398 cells will have altered response to stimuli. Because some of the differentially regulated genes were components of the glutamatergic pathway, and because we detected as much as 20% excitatory glutamatergic neurons in the mDA cultures (Fig. 1O,P), we reasoned that the increased excitability in mDA cultures may be due to nAChR activity in the DA neurons or to pre-synaptic input from other cells in culture—possibly an excitatory input. It is also possible that some TH^+^ cells co-release glutamate. Recent reports identify GABA release by DA neurons^48^,^49^. To test this prediction we differentiated iPSC into glutamatergic neurons.

Results using excitatory iN cell cultures and nicotine exposure confirm the prediction that N398 cells exhibit a more robust network activity response to nicotine specifically in excitatory neurons. This enhanced activity was confirmed by assessing the frequencies of PSCs in response to increasing nicotine exposure (Fig. 4). Results show an increased frequency of PSCs in N398 iN cells at the lowest dose tested (0.1 μM), followed by results at higher doses that were consistent with desensitization of the receptor, as was observed in a Xenopus injection model^14^. Desensitization was only found in that model with α2β4-containing nAChRs^14^ and, while other subunit mRNAs were present in our cultures, α2 and β4 mRNAs are prominent. Levels of the α5 subunit mRNA were somewhat lower in N398 mDA neurons (Fig. 1Q), which was confirmed and extended by the RNAseq study (Supplemental Table 3), so it remains possible that reduced neuronal expression of α5 may contribute to the observed nicotine sensitivity. This would agree with results from Chrna5 knockout mouse models as well, where reduced α5 subunit caused substantial behavioral effects^9^,^10^. Observations of the increased neuronal activity triggered by nicotine, supported by the increased basal frequency of PSCs and the predicted pathway changes revealed by RNAseq, leads us to conclude that the N398 variant produces neurons with increased responsiveness to nicotine followed by desensitization to subsequent exposure. This difference in response occurs at nicotine concentrations typical of heavy smokers (0.025–0.5 μM), potentially contributing to the increased risk of nicotine addiction^50^.

Since the stem cell-derived neuronal cultures are likely to be most similar to neurons present in early stages of human development, and not those in adults responding to repeated nicotine stimulation such as in addiction, we do not suggest that the altered activity found in N398 explains addiction behaviors. Instead, we believe that our results predict that naïve neurons in human brains of individuals with the N398 variant would exhibit enhanced response from initial nicotine exposure, perhaps contributing to development of addiction, followed by a lack of response, leading to an unsatisfied craving for additional stimulus. However, the use of iPSC-derived neuron cultures may be used to screen cessation therapies for smokers carrying specific genetic variants. Methods for genetic screening of smoking-related variants are already possible^51^,^52^ and tailoring cessation therapy to selected variants is effective^53^. Adding stem cell-based drug screening to this process is likely to produce even greater success. This stem cell-based approach serves as a proof-of-principle to study how addiction risk-associated gene variants may affect human neuronal functions. Continuing these studies is likely to lead to a better understanding of the impact of gene variants on human behavior and may identify strategies for intervention in addiction.

## Methods

### Human induced pluripotent stem cell culture and neural induction

Preparation of iPSC from human primary lymphocytes using Sendai viral vectors (CytoTune™, Life Technologies) has been described^24^. All biomaterials were de-identified repository specimens and therefore are exempt from human subjects regulations. Midbrain-like DA neurons were generated using a dual SMAD inhibition protocol^26^. Excitatory neurons were induced through a modified protocol^27^ where during day 5 of the protocol iNs were gently dissociated with accutase (StemCell Technologies), and (7.0 × 10^4^) were co-cultured with mouse glia (3.5 × 10^4^ cells)^54^. Electrophysiological analyses were performed following at least 30 days of differentiation and maturation. Additional details are found in the Supplemental Methods.

### Real-time RT-PCR (qPCR)

Total cellular RNA was prepared using TRIzol reagent (Invitrogen). Human-selective TaqMan™ probes were purchased from Life Technologies and PCR conditions followed the manufacturer’s recommendations. A sample of 17-week post-conception human fetal VTA RNA (a generous gift from Drs. Yuka Imamura Kawasawa and Nenad Sestan, Yale University) was used to normalize expression levels to obtain relative quantity (RQ) values. Statistical analysis was performed using R software; ANOVA was used to compare control and affected group means.

### RNA sequencing (RNAseq)

Preparation of cDNA libraries (TruSeq Kit, Illumina) and sequencing (HiSeq 2500, Illumina) were performed by RUCDR Infinite Biologics^®^ (Piscataway, NJ). Data were aligned with human genome using TopHat^55^ and analyzed with Cuffdiff and R/BioConductor using the cummeRbund package^55^,^56^. Sequencing data can be found in NIH repositories (SRA accession number SRP040275; GEO accession number GSE56398). H1 hESC-derived NSC data for days 0 and 5 are part of GEO series GSE56785^36^. Datasets for DA neuron cultures were from the following SRA accession numbers: SRR1030497, SRR1030496, SRR1030493, and SRR1030492^35^.

### Electrophysiology

Electrophysiology was performed as described previously^57^,^58^. Specific procedures are provided in the Supplemental Methods. Electrophysiological data are presented as mean ± SEM. All statistical comparisons were made using Student’s *t*-test or with general linear mixed model packages in R/BioConductor.

### Immunofluorescence

DA neurons were fixed for 15 min in 4% paraformaldehyde (Electron Microscopy Sciences) in PBS at room temperature and then incubated in blocking buffer (1X PBS, 1% Goat serum, 4% BSA) for 1 hr. Cells were permeabilized in 0.2% Triton X-100 in PBS for 10 minutes and incubated overnight with primary antibodies. Primary antibodies are listed in the Supplemental Methods. The Click-iT EdU Alexa Fluor^®^ 488 Imaging Kit was used to assess the mitotic state of TH^+^ neurons. Confocal and spinning disk epifluorescence confocal imaging analysis was performed using a Zeiss LSM700 or Olympus Axiovert 100 M confocal microscope (Carl Zeiss, Olympus). Cells were counted using Zeiss confocal microscope software (Zeiss). Ten images from one coverslip from two independent experiments were used for counting.

### Nicotine exposure

Cultures were whole cell patch clamped as described previously. Nicotine was perfused in extracellular medium in increasing amounts 0.1 μM to 6 μM in 3 minute intervals. EPSC frequencies were counted using the pClampfit software (Molecular dynamics). Frequencies were normalized to the first 30 seconds of wash buffer and dosage frequencies were averaged over 30 second intervals within first minute. Amplitudes within each 10 second interval were also averaged in the same time frame.

## Additional Information

**How to cite this article**: Oni, E. N. *et al*. Increased nicotine response in iPSC-derived human neurons carrying the CHRNA5 N398 allele. *Sci. Rep.* **6**, 34341; doi: 10.1038/srep34341 (2016).

## Supplementary Material

Supplementary Information
