# Supplementary material for: Increased nicotine response in iPSC-derived human neurons carrying the CHRNA5 N398 allele
Source: Sci Rep. 2016 Oct 4;6:34341. doi: 10.1038/srep34341 (PMC5048107; doi:10.1038/srep34341)

Supplement:

Increased nicotine response in iPSC-derived human neurons carrying the CHRNA5 N398 allele

Eileen N. Oni[1,#], Apoorva Halikere[2,#], Guohui Li[2], Alana J. Toro-Ramos[1], Mavis R. Swerdel[1], Jessica L. Verpeut[3], Jennifer C. Moore[4,5], Nicholas T. Bello[3], Laura J. Bierut[6], Alison Goate[7], Jay A. Tischfield[4,5], Zhiping P. Pang[2,4,*] and Ronald P. Hart[1,4,*]

Supplemental Methods:

iPSC. Human induced pluripotent stem cells were maintained at 37°C, 5% CO_2, 4% O_2 on Matrigel™ (BD Biosciences)-coated wells in mTeSR medium (Stem Cell Technologies). Cultures were cleaned of spontaneous differentiation material by hand-dissection. For differentiation, cells were dissociated using Accutase (Stem Cell Technologies) and plated at a density of 6.0 x 10^6 cells per well in a 60 cm Matrigel™-coated dish. Following reprogramming, the Sendai viral vectors were shown to be absent from iPSC genomic DNA by PCR and the RNA viral genome was lost from the cultures after 12-15 passages, as confirmed by qPCR (not shown).

Dopamine release via High Performance Liquid Chromatography (HPLC). After aspiration of medium, the cells were blanked using a HEPES external solution (140 mM NaCl, 5 mM KCl, 2 mM $CaCl_2$, 2 mM $MgCl_2$, 10 mM HEPES, 10 mM Glucose, Osm 36, pH 7.4) for 45 min. Medium was collected, flash frozen, and replaced with either 20 mM of KCl, or 1 mM of nicotine for 30 min. Culture medium samples were analyzed by reverse phase high performance liquid chromatography (HPLC; Dionex Ultimate 3000) with electrochemical detection (Coulochem III). An acetonitrile-based phosphate buffer mobile phase (Thermofisher, Sunnyvale, CA) was used for all experiments. The internal standard, 3,4-Dihydroxybenzylamine (DHBA), was added to all samples prior to extraction. To each sample, 375 μl of 0.1 N perchloric acid solution was added, then centrifuged at 12,000 rpm for 5 min. The supernatant was filtered using a 0.2 μm nylon disposable syringe filter prior to injection. This extraction procedure resulted in measurements of norepinephrine (NE), epinephrine (Epi), and dopamine (DA) by HPLC. Peak quantification was performed with Chromeleon 7.1 software (Thermofisher, Sunnyvale, CA).

Glial cell isolation. Forebrains were dissected from postnatal day 3-5 wild-type mice and were manually dissociated into ~0.5 mm^2 pieces in a total of 2 ml of HBSS. A total of 500 μl of 2.5% trypsin were added and dissociated tissue was incubated at 37°C for 20 min. Solution was mixed every 5 min. Using a pipette, the remaining tissue was further dissociated and passed through a 70 μM nylon mesh filter (BD Biosciences) into DMEM with 10% Fetal Bovine Serum and L-glutamine. Glia cells were passaged three times before culturing with human neuronal cells. These procedures were approved by the Rutgers University Institutional Animal Care and Use Committee (#I11-073-10).

Electrophysiology. The pipette solution for current-clamp experiments consisted of (in mM): 123 K-gluconate, 10 KCl, 1 $MgCl_2$, 10 HEPES, 1 EGTA, 0.1 $CaCl_2$, 1 K_2ATP, 0.2 Na_4GTP and 4 glucose. The pH of the solution was adjusted to 7.2 with KOH. A membrane potential of −65 to −70 mV was maintained during current-clamp recordings, and currents were injected into the cell in a step-wise manner to stimulate action potentials. For whole-cell voltage-dependent current recordings, the same internal solution as described above was used. For evaluation of synaptic function, the internal solution consisted of (in mM): 135 CsCl, 10 HEPES, 1 EGTA, 4 Mg-ATP, 0.4 Na_4GTP, and 10 QX-314, pH 7.4. The bath solution consisted of (in mM): 140 NaCl, 5 KCl, 2 $CaCl_2$, 2 $MgCl_2$, 10 HEPES, and glucose, pH 7.4. Stimulus artifacts in the recording, when stimuli were provided during evoked synaptic responses, were removed in the graphical representation of the data.

Antibodies. The following primary antibodies were used: Mouse anti-TH (Millipore, 1:1000), Mouse anti-Vglut1 (Synaptic Systems, 1:500), mouse anti-TuJ1 (Covance, 1:1000), rabbit anti-Synapsin (Abcam, 1:500), mouse anti-MAP2 (Millipore, 1:500), rabbit anti-DAT (Millipore, 1:250). After additional PBS washes, cells were incubated with fluorescently labeled secondary antibodies from Molecular probes (Alexa 488, Alexa 594, or Alexa 647) used at 1:500 dilution.

Supplemental Table 1. Subjects used in this study. All SNP genotypes were confirmed by PCR in iPSC DNA.

Cell Line	Gender	Status	rs880395	rs16969968	rs1799971	rs8192475	rs12914008	rs56218866	Group
70	Male	Affected	GG	AA	AA	GG	GG	TT	N398
67	Female	Affected	GG	AA	AA	GG	GG	TT	N398
31	Female	Affected	GG	AA	AA	GG	GG	TT	N398
37	Female	Control	GG	GG	AA	GG	GG	TT	D398
73	Female	Control	GG	GG	AA	GG	GG	TT	D398
41	Male	Control	GG	GG	AA	GG	GG	TT	D398

Supplemental Table 2. PluriTest algorithm values. As comparison, H1 hESC sample is used as a control for pluripotency and crude cryopreserved lymphocytes (CPL) as well as enriched $CD4^+$ T-cells serve as controls for differentiated cells. Samples from iPSC cell line 03, not used in this study, are included to match the source CPL and T-cell samples. Two different passages of line 70 and one from 41 are included to demonstrate pluripotency of iPSC lines used in this study. According to Müller et al. (2012), pluripotency can be identified with the pluripotency score greater than 20 and a novelty score less than 1.67. All iPSC pass these thresholds. TiPSC: T-cell-derived iPSC.

Subject	Stage	Group	Pluripotency raw	Pluripotency logit-p	Novelty	Novelty logit-p	RMSD
H1	ESC	hESC	20.863	1	1.53	0.034	0.462
03	CPL	CPL	-105.33	0	3.04	1	1.162
03	T-cell	T-cell	-93.18	0	2.862	1	0.996
03	iPSC	TiPSC	28.398	1	1.649	0.104	0.413
03	iPSC	TiPSC	32.179	1	1.657	0.113	0.424
70 (P26)	iPSC	TiPSC	28.115	1	1.519	0.03	0.399
70 (P13)	iPSC	TiPSC	28.238	1	1.507	0.027	0.433
41	iPSC	TiPSC	38.518	1	1.491	0.023	0.423

Supplemental Table 3. RNAseq data table. See linked Excel file (Supp Table 3 – D398N Diff Table.xls). Headings are: gene_id: official gene symbol (UCSC), sample_1/sample_2: names of groups being compared, status: OK if sufficient number of reads were observed for acceptable measurements, value_1/value_2: FPKM levels, log2_fold_change: the $\log_2$ of the ratio of N398/D398 FPKM values, test_stat: the test statistic from Cuffdiff, p_value: the uncorrected p-value of the comparison, q_value: the false discovery rate (FDR) corrected for multiple measurements, significant: yes/no (at 5% FDR, which is less stringent than the selection used in this study of 1% FDR).

Supplemental Table 4. Functional analysis of genes differentially expressed between D398 and N398. See linked Excel file (Supp Table 4 D398-N398 Functional Analysis.xls). Each worksheet is the result of comparing N398 Enriched or D398 Enriched gene lists using DAVID (Huang da, et al., 2009a, b). Abbreviations: GO, gene ontology; BP, biological process; CC, cellular component; MF, molecular function; KEGG, Kyoto encyclopedia of genes and genomes (pathway analysis).

Supplemental Figure 1. HPLC traces showing DA in medium after stimulation with 1 mM nicotine.

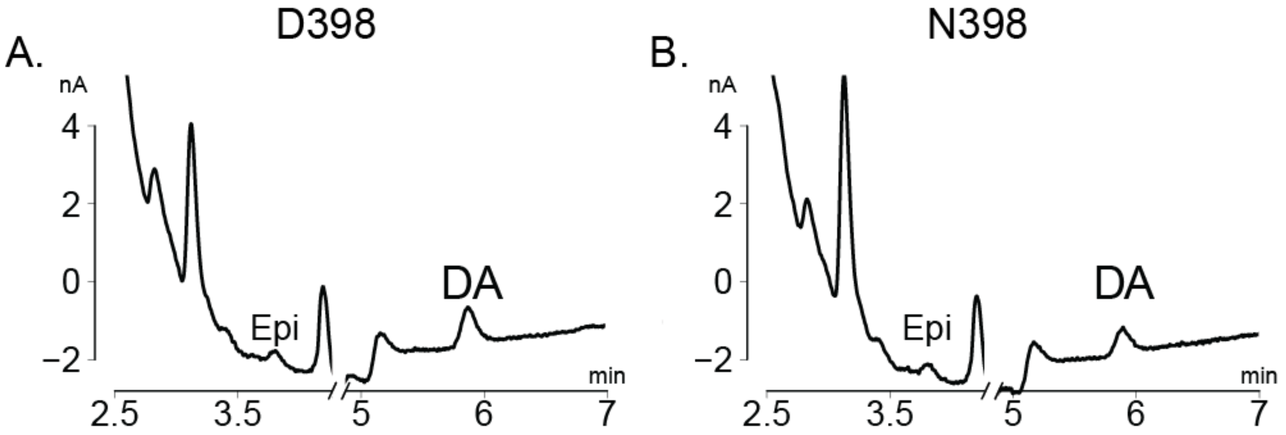

Supplemental Figure 2. Identification of alternative neuronal subtypes in mDA cultures. Cultures were stained with TH (red) and either 5HT, GAD6, or VGLUT (green).

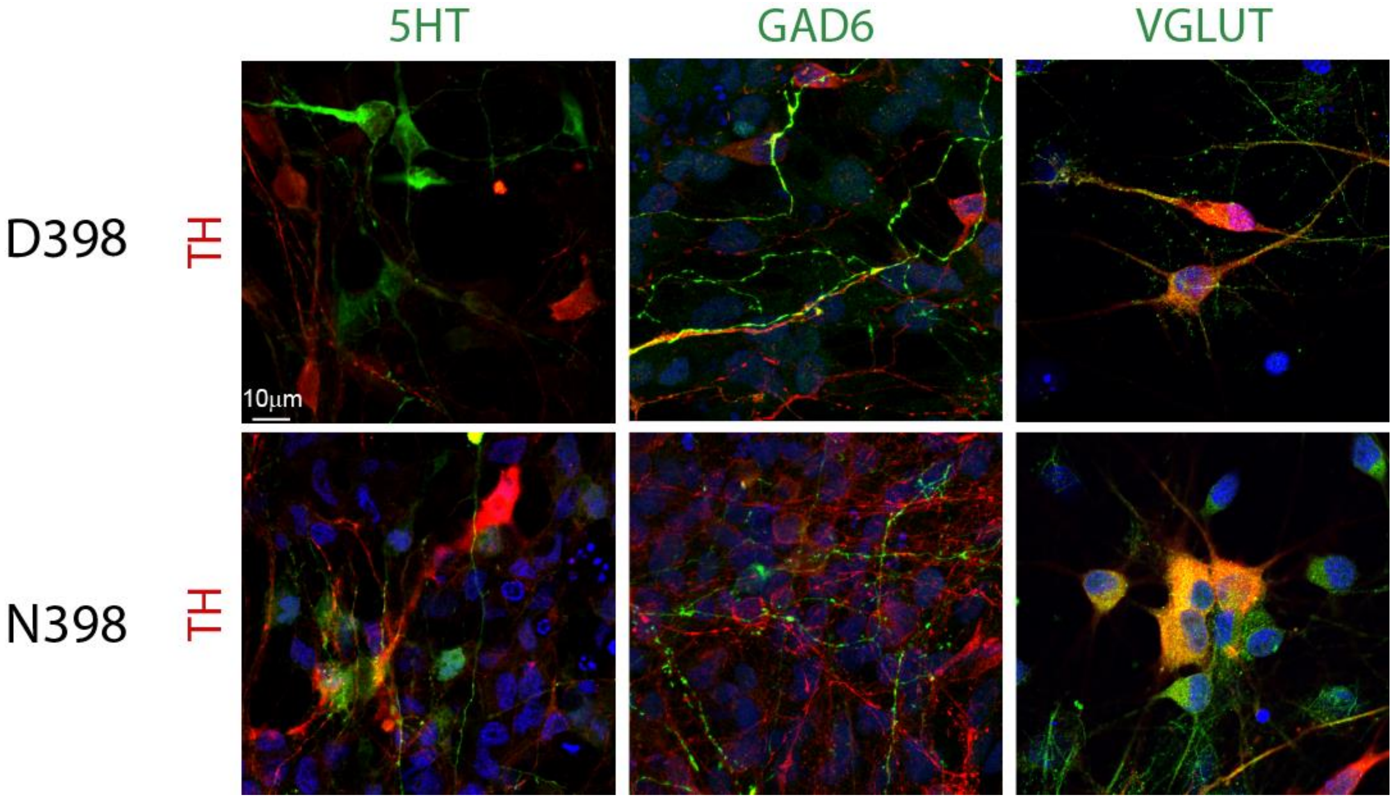

Supplemental Figure 3. N398 DA neurons exhibit increased potentiation following nicotine exposure (A) Representative traces of PSCs observed after 3 μM nicotine exposure to D398 DA neurons. (B) Pie graph showing the number of cells showing different responses to nicotine application in D398 neurons. “Potentiation, inhibition and no effect” reflect the frequencies of PSCs during the applications of nicotine, when compared to before the application of nicotine, were higher, lower or no change, respectively. (C) Representative traces and quantification of EPSCs observed after 3 μM nicotine exposure to N398 DA neurons. (D) Pie graph showing the number of cells with different responses to nicotine application in N398 neurons.

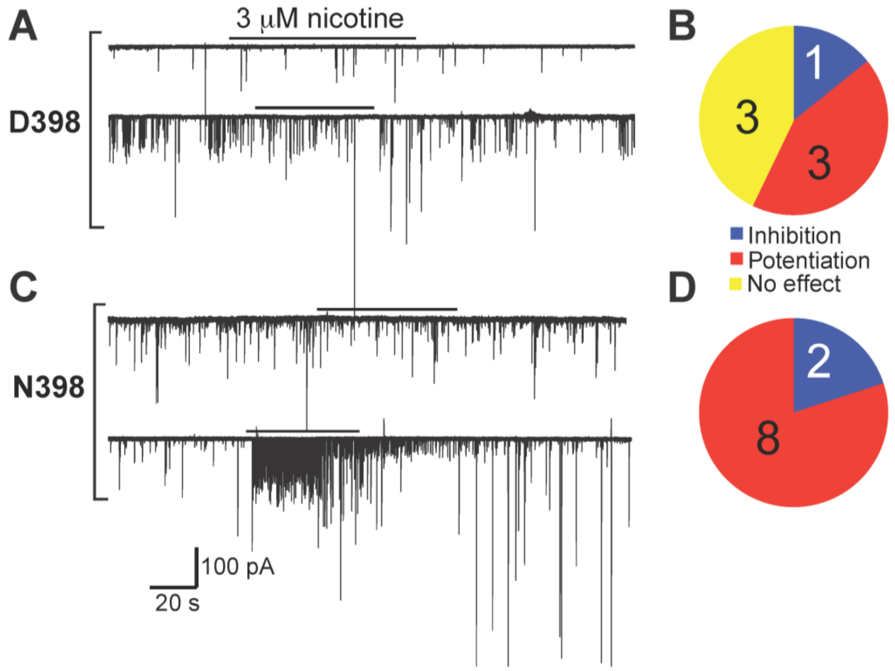

Supplemental Figure 4. Example RNA levels, in FPKM, for several representative groups of genes. FPKM levels are depicted on a log_{10} scale. (A) Cortex markers. (B) and (C) Dorsal telencephalon. (D) Cortex proliferative markers. (E)-(G) Cortical migration layer markers. (H) Selected markers from the KEGG pathway enrichment list (Fig. 3H, top bar).

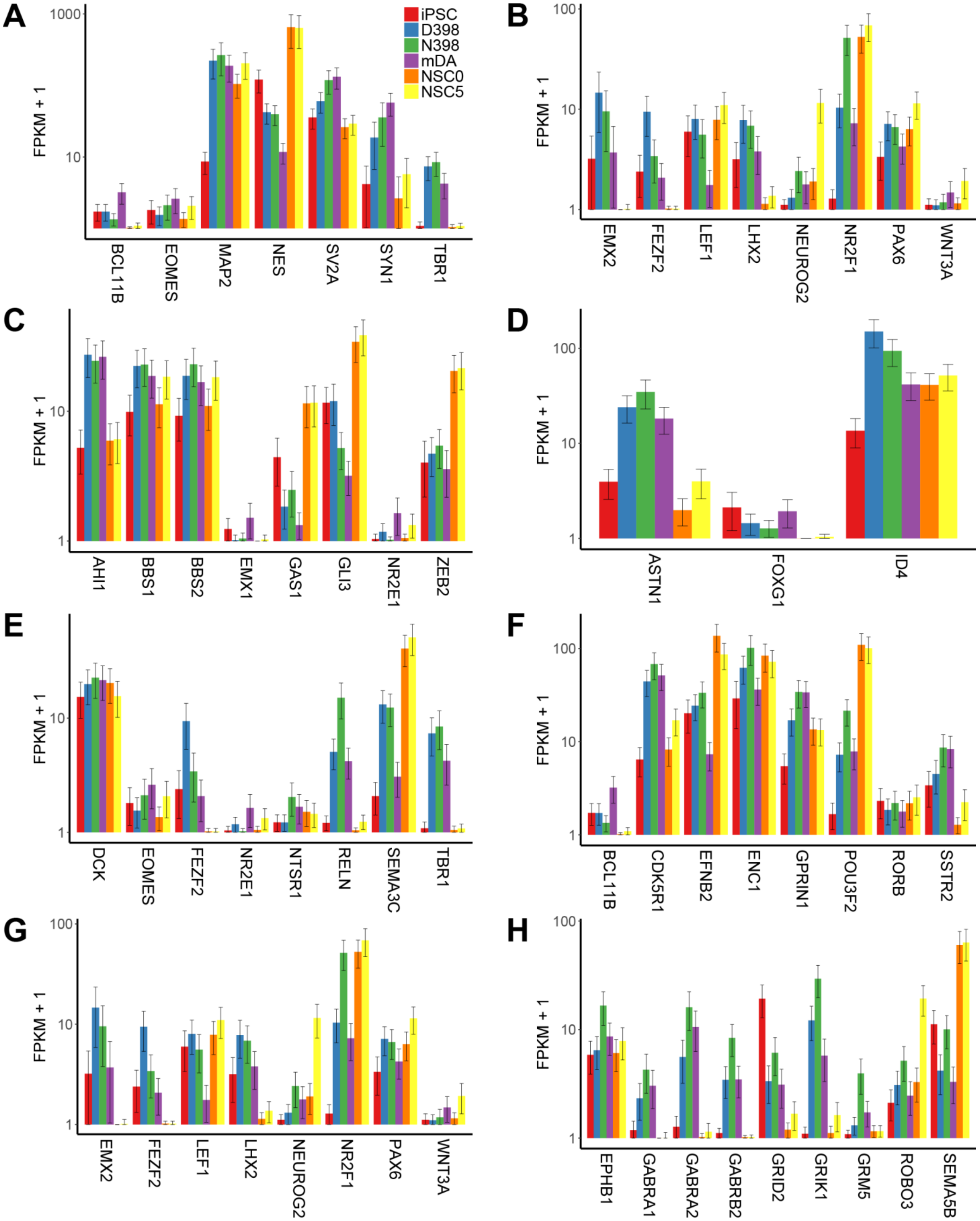

Supplemental Figure 5. Scatterplot expression patterns of gene ontology groups associated with midbrain DA neuronal differentiation, using RNAseq FPKM. In panel A, dot colors match those shown in the volcano plot in Fig. 3C. For panels B-D, blue dots denote genes within the entitled biological process group.

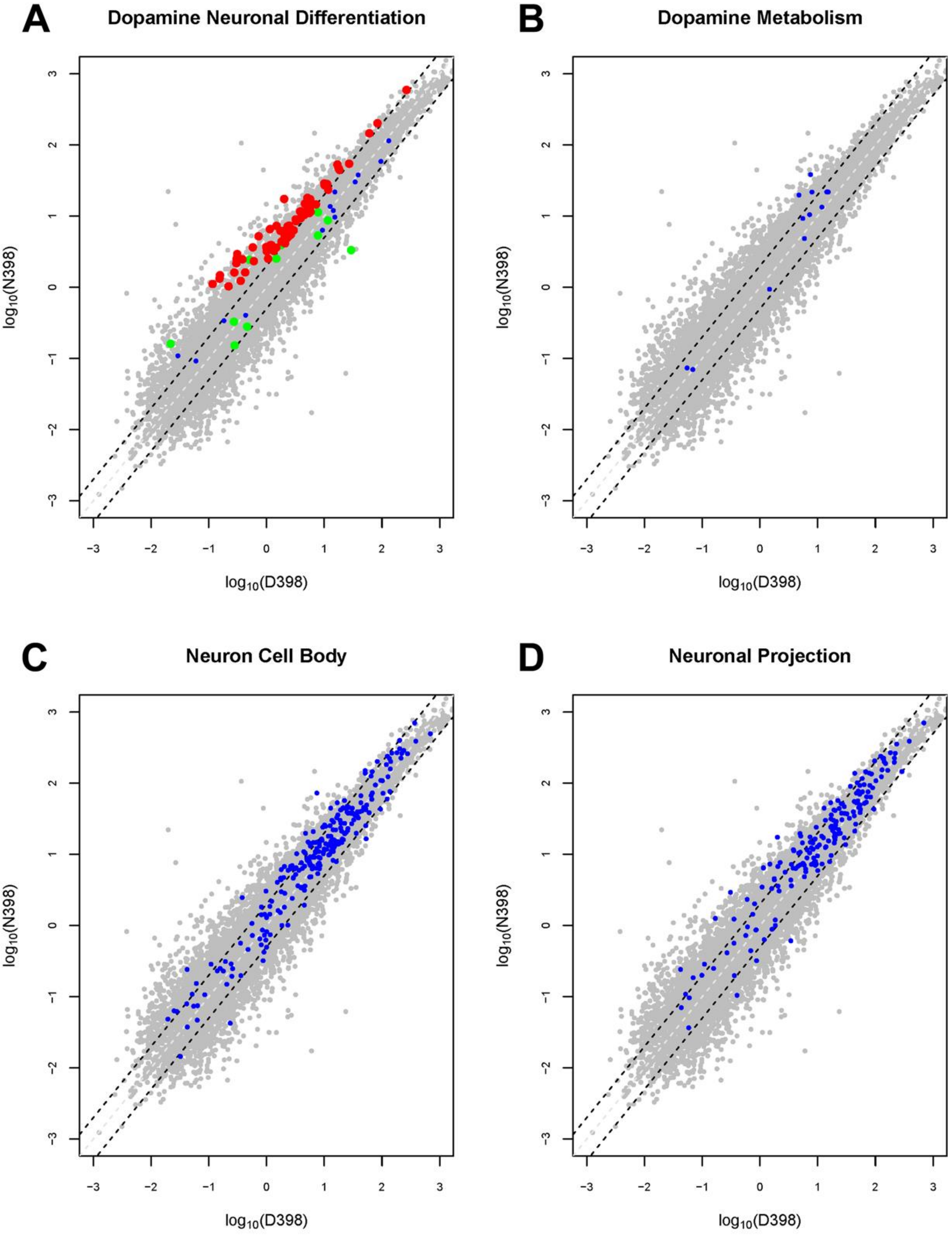

Supplement: Supplementary Information [file srep34341-s1.pdf]
